# Supplementary material for: Overestimated prediction using polygenic prediction derived from summary statistics
Source: BMC Genom Data. 2023 Sep 14;24:52. doi: 10.1186/s12863-023-01151-4 (PMC10500750; doi:10.1186/s12863-023-01151-4)
Supplement: Supplementary file 1 — Additional file 1: Table S1. Demographic characteristics [file 12863_2023_1151_MOESM1_ESM.docx]

**Table S1. Demographic characteristics**

(A) ADSP and AMP-AD

|  | ADSP | | AMP-AD | |
| --- | --- | --- | --- | --- |
|  | Cases  n = 5,687 | Controls  n = 4,606 | Cases  n = 696 | Controls  n = 437 |
| Age: years | 75.51±8.83^a^ | 87.21±4.78^a^ | 85.07±6.08 | 82.34±7.20 |
| Women No. (%) | 3,267 (57) | 2,698 (59) | 466 (67) | 267 (61) |
| *APOE* ɛ4 No. (%) | 2,405 (42) | 647 (14) | 254 (36) | 68 (15) |

(B) UK biobank

|  | Hypertension | |
| --- | --- | --- |
|  | Cases  n = 82,719 | Controls  n = 259,599 |
| Age: years | 59.70±6.91^a^ | 55.97±8.10^a^ |
| Women No. (%) | 37,844 (46) | 146,128 (56) |
| BiLEVE Axiom^b^ No. (%) | 9,860 (12) | 27,466 (11) |
| Height (m) | 1.69±0.09^a^ | 1.69±0.09^a^ |

In cases of ADSP, age means the age at onset, while in controls of ADSP, it is the age at the last assessment. The age and height of UK Biobank are those at the baseline enrollment.

^a^ means ± standard deviations

^b^ There are two DNA chip platforms: Affymetrix UK BiLEVE Axiom or Affymetrix UK Biobank Axiom array
