# Supplementary material for: Overestimated prediction using polygenic prediction derived from summary statistics
Source: BMC Genom Data. 2023 Sep 14;24:52. doi: 10.1186/s12863-023-01151-4 (PMC10500750; doi:10.1186/s12863-023-01151-4)
Supplement: Supplementary file 2 — Additional file 2: Table S2. PRS performance after excluding genetically close individuals from the test set [file 12863_2023_1151_MOESM2_ESM.docx]

**Table S2. PRS performance after excluding genetically close individuals from the test set**

|  | | AUC | | | |  | R^2^ | | | |  |
| --- | --- | --- | --- | --- | --- | --- | --- | --- | --- | --- | --- |
| PI_HAT^a^ | number^b^ | Model I | Model II | Model III^c^ | ΔAUC^c^ |  | Model I^d^ | Model II | Model III^c^ | ΔR^2c^ | *P*-value^e^ |
|  | 1133 | 64.78 | 74.03 | 80.91 | 0.069 |  | 0.069 | 0.14 | 0.26 | 0.11 | 1.51×10^–10^ |
| 1.0 | 701 | 51.27 | 80.53 | 80.70 | 0.0017 |  | –0.00142 | 0.23 | 0.24 | 0.0041 | 0.57 |
| 0.9 | 697 | 51.35 | 80.68 | 80.79 | 0.0012 |  | –0.00143 | 0.24 | 0.24 | 0.0033 | 0.67 |
| 0.8 | 697 | 51.35 | 80.68 | 80.79 | 0.0012 |  | –0.00143 | 0.24 | 0.24 | 0.0033 | 0.67 |
| 0.7 | 697 | 51.35 | 80.68 | 80.79 | 0.0012 |  | –0.00143 | 0.24 | 0.24 | 0.0033 | 0.67 |
| 0.6 | 697 | 51.35 | 80.68 | 80.79 | 0.0012 |  | –0.00143 | 0.24 | 0.24 | 0.0033 | 0.67 |
| 0.5 | 694 | 51.17 | 80.60 | 80.73 | 0.0013 |  | –0.00141 | 0.24 | 0.24 | 0.0036 | 0.65 |
| 0.4 | 692 | 51.20 | 80.67 | 80.80 | 0.0013 |  | –0.00142 | 0.24 | 0.24 | 0.0032 | 0.64 |
| 0.3 | 692 | 51.20 | 80.67 | 80.80 | 0.0013 |  | –0.00142 | 0.24 | 0.24 | 0.0032 | 0.64 |
| 0.2 | 692 | 51.20 | 80.67 | 80.80 | 0.0013 |  | –0.00142 | 0.24 | 0.24 | 0.0032 | 0.64 |
| 0.1 | 692 | 51.20 | 80.67 | 80.80 | 0.0013 |  | –0.00142 | 0.24 | 0.24 | 0.0032 | 0.64 |

^a^ Calculated after merging ADSP and AMP-AD

^b^ The number of participants of AMP-AD after excluding individuals ≥ PI_HAT

^c^ Refer to “Material and Methods” for models. The columns of Models denote the actual AUC and R^2^. ΔAUC and ΔR^2^ are obtained by subtracting AUC and R^2^ of Model II from those of Model III

^d^ The negative R^2^ values signifies a poor fit for the model

^e^ P-value for differences of AUC of Model III and Model II

Abbreviation: AUC, area under the curve
