# Supplementary material for: Overestimated prediction using polygenic prediction derived from summary statistics
Source: BMC Genom Data. 2023 Sep 14;24:52. doi: 10.1186/s12863-023-01151-4 (PMC10500750; doi:10.1186/s12863-023-01151-4)
Supplement: Supplementary file 3 — Additional file 3: Table S3. rPRS and sPRS results on AD [file 12863_2023_1151_MOESM3_ESM.docx]

**Table S3. rPRS and sPRS results on AD**

|  | Dataset | | AUC | | | |  | R^2^ | | | |  |
| --- | --- | --- | --- | --- | --- | --- | --- | --- | --- | --- | --- | --- |
|  | Discovery | test | Model I | Model II^b^ | Model III | ΔAUC^c^ |  | Model I^d^ | Model II | Model III | ΔR^2c^ | –log(p)^e^ |
| rPRS | ADSP^a^ (n=9k) | ADSP^a^ (n=1k) | 0.56±0.013 | 0.70±0.017 | 0.70±0.018 | 0.0071±0.0052 |  | 0.0098±0.0049 | 0.10±0.019 | 0.11±0.021 | 0.0077±0.0045 | 0.65±0.47 |
|  | ADSP^a^ (n=9k) | AMP-AD^f^  (n=0.7k) | 0.51±0.019 | 0.79 | 0.79±0.00091 | 0.0013±0.00091 |  | –0.0011±0.00032 | 0.20 | 0.20±0.0018 | 0.0011±0.0018 | 0.35±0.14 |
| sPRS | IGAP  (n=54k) | ADSP^a^ (n=1k) | 0.66±0.012 | 0.70±0.017 | 0.75±0.014 | 0.051±0.013 |  | 0.076±0.012 | 0.10±0.019 | 0.16±0.022 | 0.063±0.015 | 4.94±1.61 |
|  | IGAP (n=54k) | AMP-AD^f^ (n=0.7k) | 0.65 | 0.79 | 0.81 | 0.020 |  | 0.061 | 0.20 | 0.23 | 0.031 | 1.85 |
|  | IGAP (n=54k) | AMP-AD^g^ (n=1.1k) | 0.69 | 0.72 | 0.78 | 0.060 |  | 0.11 | 0.12 | 0.20 | 0.086 | 6.87 |

These results are graphically outlined in Fig. 2b and Fig. 2c of the main manuscript

After clumping within 1Mbp, all SNPs with *P* < 0.5 are used, and the number of SNPs is ~17K

The columns of Models denote the actual AUC and R^2^

^a^ Discovery and test datasets of ADSP are independent and split based on a ten-fold cross-validation

^b^ Because results from Model II depend only on the test dataset and not the discovery dataset, they are identical for a particular test dataset

^c^ The additive difference in the performance of Model III against Model II

^d^ The negative R^2^ values signifies a poor fit for the model.

^e^ p values are computed by comparing the AUC of both Model II and Model III via Delong’s methods^18^.

^f^ AMP-AD data without a close kinship with ADSP data

^g^ All AMP-AD data

Abbreviations: AUC, the area under the curve; PC, principal component; PRS, polygenic risk score
