# Supplementary material for: Overestimated prediction using polygenic prediction derived from summary statistics
Source: BMC Genom Data. 2023 Sep 14;24:52. doi: 10.1186/s12863-023-01151-4 (PMC10500750; doi:10.1186/s12863-023-01151-4)
Supplement: Supplementary file 4 — Additional file 4: Table S4. PRS performance comparisons for hypertension in UK Biobank [file 12863_2023_1151_MOESM4_ESM.docx]

**Table S4. PRS performance comparisons for hypertension in UK Biobank**

1. By the number of subjects in the discovery set

| No of Discovery dataset | AUC | | | |  | R^2^ | | | |  |
| --- | --- | --- | --- | --- | --- | --- | --- | --- | --- | --- |
|  | Model I | Model II | Model III | ΔAUC |  | Model I | Model III | Model II | ΔR^2^ | –log(p) |
| 9k | 0.51±0.0060 | 0.70±0.0044 | 0.70±0.0043 | 0.00034±0.00023 |  | 0.00025±0.00017 | 0.094±0.0041 | 0.095±0.0040 | 0.00029±0.00018 | 0.96±0.64 |
| 60k | 0.54±0.0039 | 0.70±0.0044 | 0.71±0.0042 | 0.0033±0.00047 |  | 0.0030±0.00060 | 0.094±0.0041 | 0.098±0.0041 | 0.0033±0.00051 | 6.36±0.89 |
| 300k | 0.57±0.0037 | 0.70±0.0044 | 0.72±0.0041 | 0.012±0.0014 |  | 0.011±0.0011 | 0.094±0.0041 | 0.11±0.0041 | 0.012±0.0017 | 22.69±2.65 |

The number of subjects in the test set is fixed at 34k

1. By the number of subjects in the test set

| No of Discovery dataset | AUC | | | |  | R^2^ | | | |  |
| --- | --- | --- | --- | --- | --- | --- | --- | --- | --- | --- |
|  | Model I | Model II | Model III | ΔAUC |  | Model I | Model III | Model II | ΔR^2^ | –log(p) |
| 1.7k (5%) | 0.53±0.0130 | 0.72±0.014 | 0.73±0.012 | 0.0036±0.0027 |  | 0.0030±0.0022 | 0.091±0.019 | 0.094±0.018 | 0.0032±0.0028 | 0.60±0.37 |
| 17k (50%) | 0.57±0.0054 | 0.70±0.0050 | 0.70±0.0043 | 0.0039±0.00080 |  | 0.0033±0.00080 | 0.089±0.0045 | 0.092±0.0044 | 0.0036±0.00080 | 3.98±0.78 |
| 31k (90%)^a^ | 0.57±0.0047 | 0.70±0.0041 | 0.70±0.0041 | 0.0035±0.00066 |  | 0.0031±0.00076 | 0.090±0.0038 | 0.093±0.0038 | 0.0034±0.00063 | 5.98±1.09 |

The number of subjects in the discovery set is fixed at 60K, roughly equivalent to the number of the first stage of IGAP

These results are graphically outlined in Fig. 3 of the main manuscript

After clumping within 1Mbp, all SNPs with *P* < 0.5 are used, and the number of SNPs is ~218k

The columns of Models denote the actual AUC and R^2^

^a^ The results of the 100% test set are in the second row of S2Table 2.
