# Supplementary material for: Overestimated prediction using polygenic prediction derived from summary statistics
Source: BMC Genom Data. 2023 Sep 14;24:52. doi: 10.1186/s12863-023-01151-4 (PMC10500750; doi:10.1186/s12863-023-01151-4)
Supplement: Supplementary file 5 — Additional file 5: Table S5. PRS performance comparisons for height in UK Biobank [file 12863_2023_1151_MOESM5_ESM.docx]

**Table S5. PRS performance comparisons for height in UK Biobank**

(A) By the number of subjects in the discovery set

| No of Discovery dataset* | R^2^ | | | |  |
| --- | --- | --- | --- | --- | --- |
|  | Model I | Model II | Model III | ΔR^2^ | –log(p) |
| 9k | 0.0058±0.00036 | 0.54±0.0019 | 0.54±0.0022 | 0.0054±0.00047 | 88.84±7.76 |
| 60k | 0.030±0.0021 | 0.54±0.0019 | 0.56±0.0022 | 0.028±0.0015 | ∞ |
| 300k | 0.077±0.0063 | 0.54±0.0019 | 0.61±0.0061 | 0.075±0.0056 | ∞ |

The number of subjects in the test set is fixed at 34k

(B) By the number of subjects in the test set

| No of test dataset | R^2^ | | | |  |
| --- | --- | --- | --- | --- | --- |
|  | Model I | Model III | Model II | ΔR^2^ | –log(p) |
| 1.7k (5%) | 0.032±0.0059 | 0.54±0.013 | 0.57±0.018 | 0.029±0.0037 | 23.99±2.67 |
| 17k (50%) | 0.030±0.0021 | 0.54±0.0043 | 0.56±0.0045 | 0.028±0.0014 | 234.61±11.81 |
| 31k (90%) | 0.030±0.0022 | 0.54±0.0026 | 0.56±0.0024 | 0.028±0.0015 | ∞ |

The number of subjects in the discovery set is fixed at 60k

These results are graphically outlined in Fig. 3 of the main manuscript

After clumping within 1Mbp, all SNPs with *P* < 0.5 are used, and the number of SNPs is ~221K

The columns of Models denote the actual R^2^. ΔR^2^ are obtained by subtracting R^2^ of Model II from that of Model III.
