# Supplementary material for: Overestimated prediction using polygenic prediction derived from summary statistics
Source: BMC Genom Data. 2023 Sep 14;24:52. doi: 10.1186/s12863-023-01151-4 (PMC10500750; doi:10.1186/s12863-023-01151-4)
Supplement: Supplementary file 6 — Additional file 6: Table S6. Performance comparisons between PRS and Lassosum [file 12863_2023_1151_MOESM6_ESM.docx]

**Table S6. Performance comparisons between PRS and Lassosum**

1. Hypertension

| No of Discovery dataset | ΔAUC | |  | | | ΔR^2^ | |  | |
| --- | --- | --- | --- | --- | --- | --- | --- | --- | --- |
|  | PRS | Lassosum | |  | PRS | | Lassosum | | –log(p) |
| 9k | 0.00031±0.00022 | 0.00065±0.00046 | |  | 0.00028±0.00018 | | 0.00060±0.00042 | | 1.10±0.87 |
| 60k | 0.0033±0.00047 | 0.0063±0.0018 | |  | 0.0033±0.00051 | | 0.0064±0.0017 | | 6.34±2.84 |
| 300k | 0.012±0.0014 | 0.026±0.0016 | |  | 0.012±0.0018 | | 0.028±0.0018 | | 23.61±3.18 |

1. Height

| No of Discovery dataset | ΔR^2a^ | |
| --- | --- | --- |
|  | PRS | Lassosum |
| 9k | 0.0054±0.00047 | 0.011±0.00071 |
| 60k | 0.028±0.0015 | 0.065±0.0055 |
| 300k | 0.075±0.0056 | 0.12±0.0090 |

ΔAUC and ΔR^2^ are obtained by subtracting AUC and R^2^ of Model II from those of Model III

^a^ Extra sum of squares tests used for comparing Model II and Model lII could not calculate the *P*-values for the comparison of models between PRS and Lassosum
