# Supplementary material for: Overestimated prediction using polygenic prediction derived from summary statistics
Source: BMC Genom Data. 2023 Sep 14;24:52. doi: 10.1186/s12863-023-01151-4 (PMC10500750; doi:10.1186/s12863-023-01151-4)

**Fig. S1. The number of test set subjects required to gain statistical significance (*P* < 0.01) for hypertension using UK Biobank**


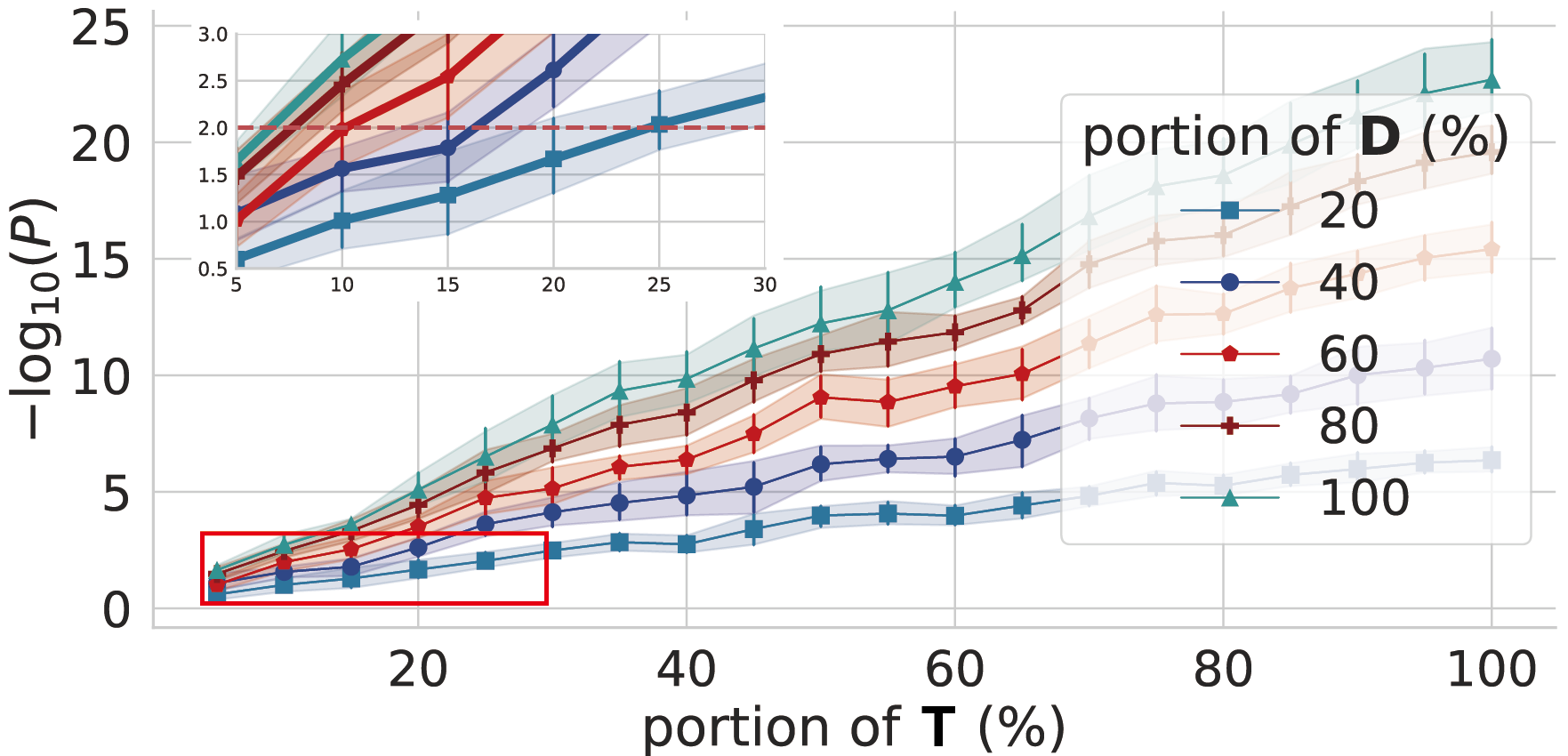

Supplement: Supplementary file 7 — Additional file 7: Fig. S1. The number of test set subjects required to gain statistical significance (P < 0.01) for hypertension using UK Biobank [file 12863_2023_1151_MOESM7_ESM.docx]
