# Supplementary material for: Overestimated prediction using polygenic prediction derived from summary statistics
Source: BMC Genom Data. 2023 Sep 14;24:52. doi: 10.1186/s12863-023-01151-4 (PMC10500750; doi:10.1186/s12863-023-01151-4)

**Fig. S2. Comparisons of PRS performance across different case:control ratios of discovery sets using hypertension phenotype of UK biobank**


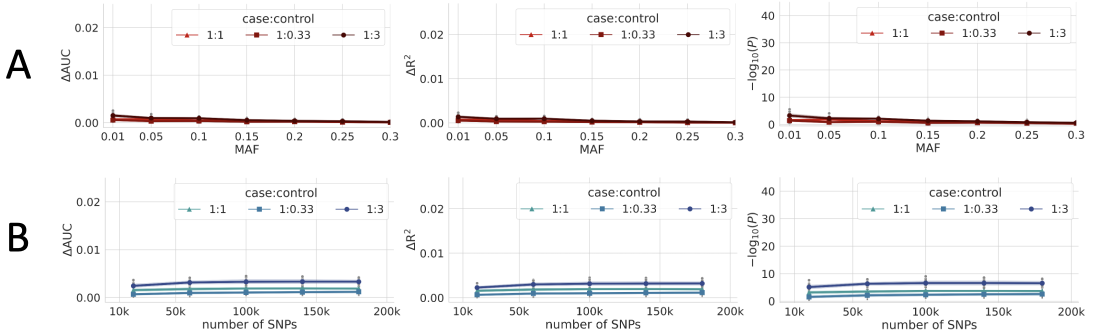

Supplement: Supplementary file 8 — Additional file 8: Fig. S2. Comparisons of PRS performance across different case:control ratios of discovery sets using hypertension phenotype of UK biobank [file 12863_2023_1151_MOESM8_ESM.docx]
