# Supplementary material for: Overestimated prediction using polygenic prediction derived from summary statistics
Source: BMC Genom Data. 2023 Sep 14;24:52. doi: 10.1186/s12863-023-01151-4 (PMC10500750; doi:10.1186/s12863-023-01151-4)
Supplement: Supplementary file 9 — Additional file 9: Supporting material: material, methods, and additional references (docx) [file 12863_2023_1151_MOESM9_ESM.docx]

**Supplementary Information**

**Overestimated prediction using polygenic prediction derived from summary statistics**

**Contents**

1. Supplementary Methods

1.1 UK Biobank data

1.2 International Genomics of Alzheimer's Project (IGAP)

1.3 ADSP and AMP-AD data

1.4 Genetic relatedness

1.5 Data processing and genotype QC of the UK Biobank

1.6 Data processing and genotype QC of the ADSP study

1.7 Data processing and genotype QC of the AMP-AD study

1.8 Imputation and selection of SNPs

1. References

**1. Supplementary Methods**

**1.1 UK Biobank data**

The UK Biobank recruited approximately 500,000 individuals in 22 centers in the United Kingdom from 2006 to 2010 [1]. The recruited people ranged in age from 38- to 73 years old. Extensive phenome information for laboratory findings, physical measurements, health records, and questionnaires about health and lifestyles were collected. We use the self-reported hypertension status and the standing height at the baseline enrollment. Detailed descriptions are in the previous landmark papers of UK Biobank [1, 2]. Data are available for download from www.ukbiobank.ac.uk.

**1.2 International Genomics of Alzheimer's Project (IGAP)**

Stage 1 of IGAP used the four previously published Genome-Wide Association Study (GWAS) datasets: EADI (European Alzheimer's Disease Initiative) [3, 4], ADGC (Alzheimer’s Disease Genetics Consortium) [5], CHARGE (The Cohorts for Heart and Aging Research in Genomic Epidemiology Consortium) [6], and GERAD (Genetic and Environmental Risk in AD Consortium) [7]. Stage 1 includes 17,008 Alzheimer's disease (AD) cases and 37,154 controls. Stage 2 includes 8,572 AD cases and 11,312 controls. All participants are individuals of European ancestry. Finally, a meta-analysis is performed, combining results from Stages 1 and 2 [8]. In this study, the summary statistics of Stage 1 were downloaded from http://web.pasteur-lille.fr/en/recherche/u744/igap/igap_download.php.

**1.3 Alzheimer's Disease Sequencing Project (ADSP) and AMP-AD data**

ADSP is a whole-exome sequencing study that consists of 5,844 AD cases and 4,767 controls. AMP-AD is a whole-genome sequencing study with 955 AD cases and 821 controls. The participants of ADSP are derived from 24 cohorts of AD Genetics Consortium and cohorts for Heart & Aging Research in Genomic Epidemiology. All cases meet the NINCDS-ADRDA (National Institute of Neurological and Communicative Diseases and Stroke/Alzheimer's Disease and Related Disorders Association) criteria for possible, probable, or definite AD with documented age at onset or age at death (for pathologically verified cases), as well as *APOE* (Apolipoprotein E) genotyping. All controls are at least 60 years old and free of dementia by direct or documented cognitive assessment. Additional descriptions of the sample selection criteria for the ADSP samples can be found elsewhere [9]. The AMP-AD study consists of three genetics studies: The Mayo Clinic AD Genetics Studies [10], Mount Sinai Brain Bank (MSBB) study [11], and Religious Orders Study and Memory and Aging Project (ROSMAP) Study [12].

**1.4 Genetic relatedness**

We exclude participants with closer kinship within the third degree using the kinship matrix precalculated and deposited on the UK Biobank webpage. The kinship matrix has been calculated using KING [13]. For AD genetic studies, we obtained PI_HAT values using the genome function of PLINK (v1.9) after merging the common variants (MAF≥1%) of ADSP and AMP-AD data [14]. Before merging, the participants with close relatedness (PI_HAT > 0.1 in ADSP and PI_HAT > 0.2 in AMP-AD) within the studies are excluded. Subjects from AMP-AD are regarded as genetically identical to those in ADSP when PI_HAT=1.0 (n=432) and closely related when PI_HAT>0.2 (n=441).

**1.5 Data processing and genotype QC of the UK Biobank**

Using the UK BiLEVE array and UK Biobank axiom array, 488,377 participants’ samples are successfully genotyped. We involve only individuals with white-British ancestry in this study. We exclude the third-degree related individuals (kinship coefficients < 0.0442). Also, individuals are excluded when there is a sex mismatch or sex chromosome aneuploidy, or there are outliers in heterozygosity and missing rates. Finally, a total of 342,318 individuals who have hypertension and height phenotypes are used in the study.

**1.6 Data processing and genotype QC of the ADSP study**

Sequencing for the ADSP project was performed in three centers: the Broad Institute, Human Genome Sequencing Center at the Baylor College of Medicine, and McDonnell Genome Institute at Washington University in St. Louis. The quality control (QC) step of the sequenced whole-exome sequencing (WES) data followed the GATK (genomic analysis toolkit) best practices. The inclusion criteria for variants are mapping score ≥0.8, high (≥10) mean read depth, in-range VR/DP (variant read/total read depth ratio ≥0.25 and ≤0.75), and allele balance (AB) scores ranging from 0.3 to 0.7. The exclusion criteria for variants are high (≥20%) missingness, very high (≥500) read depth, excess heterozygosity (for MAF<0.2, |z|>1.22; for MAF≥0.2, |z|>5SD), monomorphic variants, variants with controls’ deviation of Hardy-Weinberg Equilibrium (*P* < 5.0×10^–5^, only in MAF>0.1%), and variants with missingness test *P* < 1.0×10^–8^ between cases and controls. The exclusion criteria for individuals are excess (>6SD) private variants, low (≤80%) call rate, high (>6SD) Ti/Tv ratio, high (>6SD) heterozygosity-to-homozygosity ratio, and high (>500) mean read depth. Finally, 10,907 subjects with 1,508,430 variants in a case-control study remained. We exclude outliers using the leading two principal components (PCs). Among them, we analyzed the data of 10,294 European participants who have phenotypic information.

**1.7 Data processing and genotype QC of the AMP-AD study**

After downloading whole-genome sequencing data (WGS) from https://adknowledgeportal.synapse.org/, we select variants with high tranche sensitivity (>99.9%) and their quality (≥30). Additionally, the genotypes with high-quality depth (QD≥5 for indels and QD≥2 for single nucleotide polymorphisms [SNPs]), in-range AB (>0.3 and <0.7), high (≥5) mean depth, and low (≤5%) missingness are selected. We exclude variants with maximal read depth >5SD and those in the low complexity regions. We discard variants with high significance (*P* < 1.0×10^–8^) in the missingness tests (between cases and control; different studies) and deviation of Hardy-Weinberg Equilibrium (*P* < 1.0×10^–6^) in controls. The individuals with high (>10%) missing genotypes are excluded. We check sex with PLINK (v1.9) [14].

**1.8 Imputation and selection of SNPs**

Version 3 imputation genotype data are downloaded from www.ukbiobank.ac.uk. Before imputation, the genotyped data are phased using a modified version of the SHAPEIT2 algorithm [15]. The multiallelic or rare (MAF ≤1%) SNPs are removed before phasing. Genotype imputation is performed using IMPUTE2 algorithms [16]. The UK10K haplotype and HRC reference panels are combined for the imputation reference set [17]. The imputation INFO cutoff scores differ according to MAFs (Info>0.3 for MAF >3%; Info>0.6 for MAF 1-3%; Info>0.8 for MAF 0.5-1%; Info>0.9 for MAF 0.1-0.5%) [1]. We filter out SNPs with MAF < 1% and deviation of Hardy-Weinberg Equilibrium (*P* < 1.0×10^–8^).

We use imputed data to increase the common variants between ADSP and AMP-AD data. After selecting variants with MAF ≥ 1% and call rate > 95% in addition to genotype QC, we perform imputation using the Michigan imputation server (<https://imputationserver.sph.umich.edu/>) with multi-population reference panels of Phase III 1000 genomes and default parameters. After imputation, we discard variants with MAF < 1% and R^2^ < 0.3.

**References**

1. Bycroft C, Freeman C, Petkova D, Band G, Elliott LT, Sharp K, et al. The UK Biobank resource with deep phenotyping and genomic data. Nature. 2018;562(7726):203-9. Epub 2018/10/12. doi: 10.1038/s41586-018-0579-z. PubMed PMID: 30305743; PubMed Central PMCID: PMCPMC6786975.

2. Collins R. What makes UK Biobank special? Lancet. 2012;379(9822):1173-4. Epub 2012/04/03. doi: 10.1016/S0140-6736(12)60404-8. PubMed PMID: 22463865.

3. Group CS. Vascular factors and risk of dementia: design of the Three-City Study and baseline characteristics of the study population. Neuroepidemiology. 2003;22(6):316-25. Epub 2003/11/06. doi: 10.1159/000072920. PubMed PMID: 14598854.

4. Dreses-Werringloer U, Lambert JC, Vingtdeux V, Zhao H, Vais H, Siebert A, et al. A polymorphism in CALHM1 influences Ca2+ homeostasis, Abeta levels, and Alzheimer's disease risk. Cell. 2008;133(7):1149-61. Epub 2008/07/01. doi: 10.1016/j.cell.2008.05.048. PubMed PMID: 18585350; PubMed Central PMCID: PMCPMC2577842.

5. Jun G, Naj AC, Beecham GW, Wang LS, Buros J, Gallins PJ, et al. Meta-analysis confirms CR1, CLU, and PICALM as alzheimer disease risk loci and reveals interactions with APOE genotypes. Arch Neurol. 2010;67(12):1473-84. Epub 2010/08/11. doi: 10.1001/archneurol.2010.201. PubMed PMID: 20697030; PubMed Central PMCID: PMCPMC3048805.

6. Psaty BM, O'Donnell CJ, Gudnason V, Lunetta KL, Folsom AR, Rotter JI, et al. Cohorts for Heart and Aging Research in Genomic Epidemiology (CHARGE) Consortium: Design of prospective meta-analyses of genome-wide association studies from 5 cohorts. Circ Cardiovasc Genet. 2009;2(1):73-80. Epub 2009/12/25. doi: 10.1161/CIRCGENETICS.108.829747. PubMed PMID: 20031568; PubMed Central PMCID: PMCPMC2875693.

7. Harold D, Abraham R, Hollingworth P, Sims R, Gerrish A, Hamshere ML, et al. Genome-wide association study identifies variants at CLU and PICALM associated with Alzheimer's disease. Nat Genet. 2009;41(10):1088-93. Epub 2009/09/08. doi: 10.1038/ng.440. PubMed PMID: 19734902; PubMed Central PMCID: PMCPMC2845877.

8. Lambert JC, Ibrahim-Verbaas CA, Harold D, Naj AC, Sims R, Bellenguez C, et al. Meta-analysis of 74,046 individuals identifies 11 new susceptibility loci for Alzheimer's disease. Nat Genet. 2013;45(12):1452-8. Epub 2013/10/29. doi: 10.1038/ng.2802. PubMed PMID: 24162737; PubMed Central PMCID: PMCPMC3896259.

9. Beecham GW, Bis JC, Martin ER, Choi SH, DeStefano AL, van Duijn CM, et al. The Alzheimer's Disease Sequencing Project: Study design and sample selection. Neurol Genet. 2017;3(5):e194. Epub 2017/12/01. doi: 10.1212/NXG.0000000000000194. PubMed PMID: 29184913; PubMed Central PMCID: PMCPMC5646177.

10. Allen M, Carrasquillo MM, Funk C, Heavner BD, Zou F, Younkin CS, et al. Human whole genome genotype and transcriptome data for Alzheimer's and other neurodegenerative diseases. Sci Data. 2016;3:160089. Epub 2016/10/12. doi: 10.1038/sdata.2016.89. PubMed PMID: 27727239; PubMed Central PMCID: PMCPMC5058336 Immunotherapy: Chair, Data Monitoring Committee. Hoffman-La Roche, Inc.: Consultant. Merck, Inc.: Consultant. Genentech, Inc.: Consultant. Biogen, Inc.: Consultant. Eli Lilly & Co.: Consultant. N.R.G.-R. has multicenter treatment study grants from Lilly and TauRx and consulted for Cytox. N.E.-T. has consulted for Cytox. The remaining authors declare no competing financial interests.

11. Wang M, Beckmann ND, Roussos P, Wang E, Zhou X, Wang Q, et al. The Mount Sinai cohort of large-scale genomic, transcriptomic and proteomic data in Alzheimer's disease. Sci Data. 2018;5:180185. Epub 2018/09/12. doi: 10.1038/sdata.2018.185. PubMed PMID: 30204156; PubMed Central PMCID: PMCPMC6132187.

12. De Jager PL, Ma Y, McCabe C, Xu J, Vardarajan BN, Felsky D, et al. A multi-omic atlas of the human frontal cortex for aging and Alzheimer's disease research. Sci Data. 2018;5:180142. Epub 2018/08/08. doi: 10.1038/sdata.2018.142. PubMed PMID: 30084846; PubMed Central PMCID: PMCPMC6080491.

13. Manichaikul A, Mychaleckyj JC, Rich SS, Daly K, Sale M, Chen WM. Robust relationship inference in genome-wide association studies. Bioinformatics. 2010;26(22):2867-73. Epub 2010/10/12. doi: 10.1093/bioinformatics/btq559. PubMed PMID: 20926424; PubMed Central PMCID: PMCPMC3025716.

14. Chang CC, Chow CC, Tellier LC, Vattikuti S, Purcell SM, Lee JJ. Second-generation PLINK: rising to the challenge of larger and richer datasets. Gigascience. 2015;4:7. Epub 2015/02/28. doi: 10.1186/s13742-015-0047-8. PubMed PMID: 25722852; PubMed Central PMCID: PMCPMC4342193.

15. O'Connell J, Sharp K, Shrine N, Wain L, Hall I, Tobin M, et al. Haplotype estimation for biobank-scale data sets. Nat Genet. 2016;48(7):817-20. Epub 2016/06/09. doi: 10.1038/ng.3583. PubMed PMID: 27270105; PubMed Central PMCID: PMCPMC4926957.

16. Howie B, Marchini J, Stephens M. Genotype imputation with thousands of genomes. G3 (Bethesda). 2011;1(6):457-70. Epub 2012/03/03. doi: 10.1534/g3.111.001198. PubMed PMID: 22384356; PubMed Central PMCID: PMCPMC3276165.

17. Huang J, Howie B, McCarthy S, Memari Y, Walter K, Min JL, et al. Improved imputation of low-frequency and rare variants using the UK10K haplotype reference panel. Nat Commun. 2015;6:8111. Epub 2015/09/15. doi: 10.1038/ncomms9111. PubMed PMID: 26368830; PubMed Central PMCID: PMCPMC4579394.
