# Supplementary material for: Overestimated prediction using polygenic prediction derived from summary statistics
Source: BMC Genom Data. 2023 Sep 14;24:52. doi: 10.1186/s12863-023-01151-4 (PMC10500750; doi:10.1186/s12863-023-01151-4)
Supplement: Supplementary file 10 — Supplementary Material 10 [file 12863_2023_1151_MOESM10_ESM.docx]

**Supplementary Information**

**Additional file 1 : Table S1.** Demographic characteristics (docx)

**Additional file 2 : Table S2.** PRS performance after excluding genetically close individuals from the discovery set (docx)

**Additional file 3 : Table S3.** Results of rPRSs and sPRS (docx)

**Additional file 4 : Table S4.** PRS performances of hypertension in UK Biobank (docx)

**Additional file 5 : Table S5.** PRS performances of height in UK Biobank (docx)

**Additional file 6 : Table S6.** Comparison between two PRSs methods (docx)

**Additional file 7 : Supplementary Figure 1.** Least number of test set subjects required to gain statistical significance (P<0.01) for hypertension using UK Biobank. P-values are obtained by comparing AUC of Model II and Model III. The five lines denote different sizes of the discovery set (D), where 100% corresponds to 300k. The x-axis shows the size of the test set (T) where 100% is 34k. The small plot in the left upper quadrant shows a higher magnification of the red box area, in which the significance threshold is drawn using a dotted red line (docx)

**Additional file 8 : Supplementary Figure 2.** Different results according to case:control ratios. We fixed the number of cases to that of the IGAP study. Three distinct lines represent different case:control ratios—1:0.33, 1:1, and 1:3. (A) The X-axis corresponds to MAF intervals. MAF intervals are split into seven categories—0.01–0.05, 0.05–0.10, 0.10–0.15, 0.15–0.20, 0.20–0.25, 0.25–0.30, and > 0.3. (B) The 'number of SNPs' on the X-axis indicates the count of LD-pruned SNPs, selected based on the lowest P-value thresholds (docx)

**Additional file 9 : Supporting material: material, methods, and additional references** (docx)
